# A full-length transcriptome and gene expression analysis reveal genes and molecular elements expressed during seed development in *Gnetum luofuense*

**DOI:** 10.1186/s12870-020-02729-1

**Published:** 2020-11-23

**Authors:** Nan Deng, Chen Hou, Boxiang He, Fengfeng Ma, Qingan Song, Shengqing Shi, Caixia Liu, Yuxin Tian

**Affiliations:** 1 Hunan Academy of Forestry, Changsha, Hunan, No.658 Shaoshan Road, Tianxin District, Changsha, 410004 China; 2 Hunan Cili Forest Ecosystem State Research Station, Cili, Changsha, 410004 Hunan China; 3 Guangdong Academy of Forestry, Guangzhou, 510520 China; 4 Guangdong Provincial Key Laboratory of Silviculture, Protection and Utilization, Guangdong Academy of Forestry, Guangzhou, 510520 China; 5 State Key Laboratory of Tree Genetics and Breeding, Research Institute of Forestry, Chinese Academy of Forestry, No. 1 Dongxiaofu, Xiangshan Road, Haidian, Beijing, 100091 China

**Keywords:** Gnetales, Full-length transcriptome, Functional genes, Seed, lncRNA

## Abstract

**Background:**

*Gnetum* is an economically important tropical and subtropical gymnosperm genus with various dietary, industrial and medicinal uses. Many carbohydrates, proteins and fibers accumulate during the ripening of *Gnetum* seeds. However, the molecular mechanisms related to this process remain unknown.

**Results:**

We therefore assembled a full-length transcriptome from immature and mature *G. luofuense* seeds using PacBio sequencing reads. We identified a total of 5726 novel genes, 9061 alternative splicing events, 3551 lncRNAs, 2160 transcription factors, and we found that 8512 genes possessed at least one poly(A) site. In addition, gene expression comparisons of six transcriptomes generated by Illumina sequencing showed that 14,323 genes were differentially expressed from an immature stage to a mature stage with 7891 genes upregulated and 6432 genes downregulated. The expression of 14 differentially expressed transcription factors from the MADS-box, Aux/IAA and bHLH families was validated by qRT-PCR, suggesting that they may have important roles in seed ripening of *G. luofuense*.

**Conclusions:**

These findings provide a valuable molecular resource for understanding seed development of gymnosperms.

**Supplementary Information:**

The online version contains supplementary material available at 10.1186/s12870-020-02729-1.

## Background

**Fig. 1:**
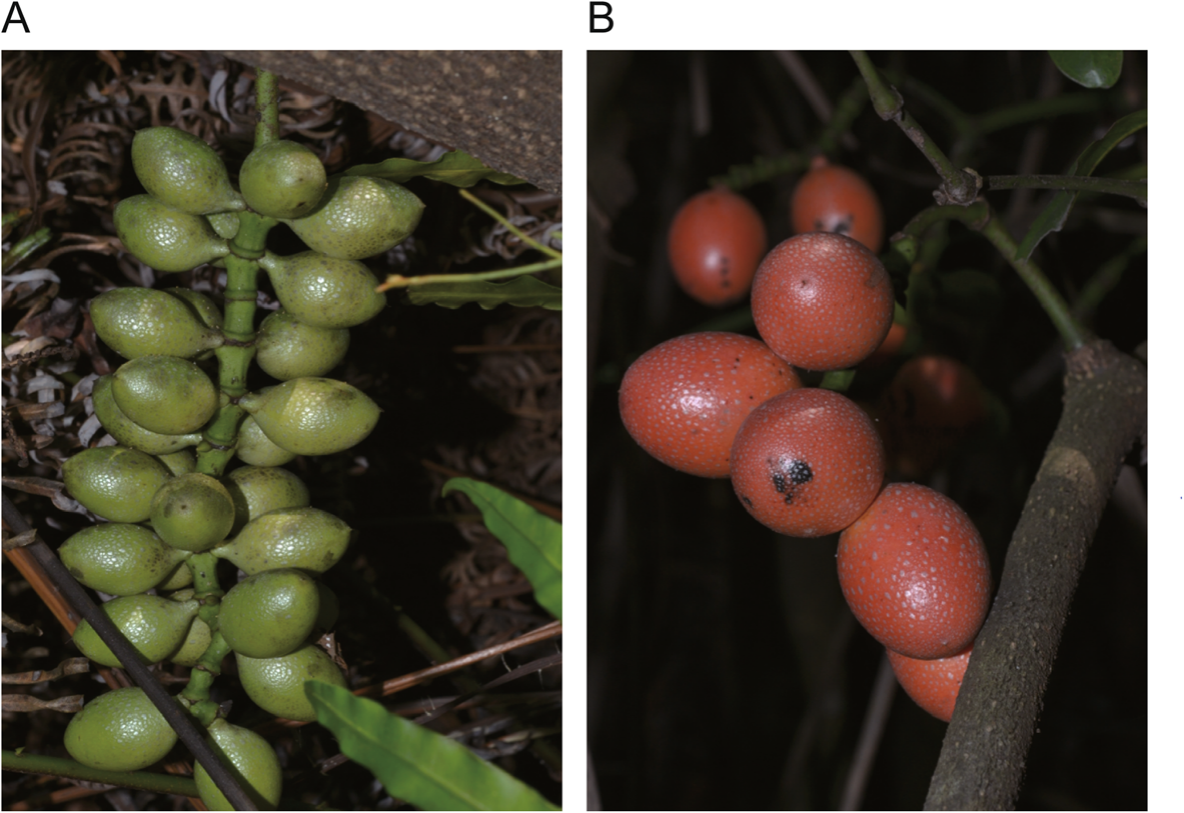
Two developmental stages of *G. lufouense* seeds. **a** Immature seeds. **b** Mature seeds

*Gnetum* is a genus of tropical and subtropical gymnosperm trees and shrubs distributed in South America, eastern Africa, and Asia [1]. *Gnetum* possesses remarkable economic potential for dietary and industrial use: its leaves are used as a vegetable, its stems and bark are made into string, nets and paper, and its seeds are used in oil and drinks. A *Gnetum* seed originates from a female reproductive unit that is produced on the collar involucre of a female strobilus [1, 2]. A *Gnetum* seed is composed of three layers of envelopes, the outermost of which gives rise to a seed coat—aril [3, 4]. *Gnetum* seeds are rich in a variety of chemicals, such as carbohydrates, proteins and fibers [5, 6]. The primary metabolism (e.g. carbohydrate metabolism) is probably associated with seed ripening process (during which the aril color changes from green to red, Fig. 1) in *Gnetum*, but the molecular mechanisms that underlie the process have not been carefully investigated.

Previous investigations of transcription factors (TFs) provide valuable insight into the molecular mechanisms of reproductive organ development in *Gnetum*. MADS-box genes, comprising type I and type II MADS-box genes, encode essential transcription factors that regulate reproductive organ development in seed plants [7]. Previous work has shown that type II MADS-box *AG*-like and *TM8*-like genes are highly expressed in *G. luofuense* seeds [8]. In addition, *Aux/IAA* genes participate in the development of various organs in seed plants by responding to the hormone auxin/indole-3-acetic acid [9, 10]. A recent study showed that six *Aux/IAA* genes are involved in the development of female strobili in *G. luofuense* [11]. Another study reported that *bHLH* genes facilitate the development of *G. luofuense* leaves [11]. Moreover, bHLH and MYB TFs are able to form a complex that regulates stamen development and seed production [12]. Therefore, genes that encode MADS-box, Aux/IAA and bHLH TFs may play essential roles in *Gnetum* seed ripening, and these possibilities require further examination.

In addition to key genes/TFs, other molecular mechanisms that regulate male strobilus development in *G. luofuense* are also noteworthy. For example, previous studies have shown that *G. luofuense* uses alternative splicing (AS) and alternative polyadenylation (APA) to enrich transcriptome complexity during the development of leaves and female strobili [11]. AS has been proposed as an essential modulator of development in eukaryotic organisms [13, 14]. Besides, APA facilitates the stability, translation and localization of target RNAs by generating varied isoforms with different coding sequences or 3′ UTRs [15]. There have been few investigations of AS and APA in gymnosperms, but such studies are much more abundant in angiosperms (e.g. [16–21]). In addition, long noncoding RNAs (lncRNAs), which possess at least 200 nucleotides, may also play a role in the regulation of *Gnetum* seed development. LncRNAs take part in transcriptional and post-transcriptional gene regulation in almost all eukaryotic organisms [22–24]. The presence of lncRNAs has only been reported in the leaves of *Gingko biloba* L. [25, 26] and in the leaves and female strobili of *G. luofuense* [11]. To date, little attention has been paid to lncRNAs in gymnosperms [11, 27].

To investigate AS, APA and lncRNAs, PacBio sequencing provides better performance than Illumina sequencing, it is because single-molecule transcriptome sequencing provides greater sequence completeness with regard to the 5′ and 3′ ends of cDNA molecules, higher accuracy for the identification of alternative isoforms, and increased power to distinguish RNA haplotypes [11, 16, 28]. Therefore, in the present study, we generated a full-length transcriptome from two developmental stages (immature and mature) of *G. luofuense* seeds using the reference genome of *G. luofuense* (=*G. montanum*) [29]. AS, APA, lncRNAs and relevant TFs were investigated using the single-molecule data. In addition, we generated separate transcriptomes for the two seed developmental stages using Illumina RNA sequencing to uncover key genes that regulate the seed ripening process in *Gnetum*.

## Results

### PacBio sequencing and error correction

The full-length transcriptome of mature and immature *G. luofuense* seeds comprised a total of 12,869,707 subreads (19.81 Gb) with an average length of 1540 bp (Table S1, Fig. S1A). After self-correction with an accuracy value of ROIs > 0.8, 384,042 circular consensus sequences (CCSs) with an average length of 1919 bp were generated, of which full-length, non-chimeric (FLNC) reads accounted for 81% (312,444, Fig. S1B). The FLNC reads were clustered using the ICE algorithm, and non-FLNC reads were polished. The FLNC reads and polished non-FLNC reads were merged, yielding 165,883 polished consensus isoforms ranging from 167 to 13,816 bp in length (Fig. S1C). The 165,883 polished consensus reads were further corrected using Illumina sequencing data with LoRDEC software. The mean length and N50 and N95 values changed slightly after correction (Table S2).

### Genome mapping and novel gene detection

The corrected polished consensus reads were mapped to the *G. luofuense* reference genome using GMAP. 162,887 (98.19%) reads were mapped to the reference (Fig. S1D); of these, 63,049 uniquely mapped reads (38.01% of total mapped reads) were mapped to the positive strand of the reference genome, 60,292 uniquely mapped (36.35%) reads were mapped to the negative strand, 39,546 (23.84%) were multiply mapped reads, and 2996 (1.81%) reads were unmapped. The mapping density on each scaffold of *G. luofuense* genome was shown in Fig. S1E. Over 98% of the mapped reads showed similarity to the reference genome, and coverage values of the mapped reads were all above 80% (Fig. S1F). After deleting the unmapped and redundant reads, 41,151 reads remained, of which 7899 were novel isoforms of known genes and 5726 reads were from novel genes.

### Annotation and classification of novel genes

The 5726 novel genes were annotated by searching against six databases—NCBI NR, KEGG, GO, SwissProt, KOG, and Pfam. A total of 4099 novel genes were annotated, of which 2588 were annotated in the NR database (Table S3). Five species—*Picea sitchensis* (649 genes), *Amborella trichopoda* (116), *Vitis vinifera* (88), *Elaeis guineensis* (80), and *Nelumbo nucifera* (61)—produced the largest numbers of hits to the *G. luofuense* novel genes (Fig. S2A). Two thousand four hundred eighty-seven novel genes were annotated with KEGG pathways (Table S3), and the most enriched pathways were “signal transduction” (169 genes), “carbohydrate metabolism” (83 genes), and “translation” (69 genes, Fig. S2B). GO analysis classified 2069 genes into three categories: “biological process”, “cellular components” and “molecular functions” (Fig. S2C). Novel genes classified in the biological process category were mainly annotated with the terms “metabolic process” (1052), “cellular process” (1037), and “single-organism process” (581). Novel genes classified in the cellular component category were mainly annotated with the terms “cell” (519), “cell part” (519), and “membrane” (367). Novel genes classified in the molecular function category were mainly annotated with the terms “binding” (1192), “catalytic activity” (942), and “transporter activity” (132). One thousand nine hundred thirty genes, 1315 genes and 2069 genes were annotated with the Swiss Prot, KOG and Pfam databases, respectively (Table S3).

### AS and APA analysis

**Fig. 2:**
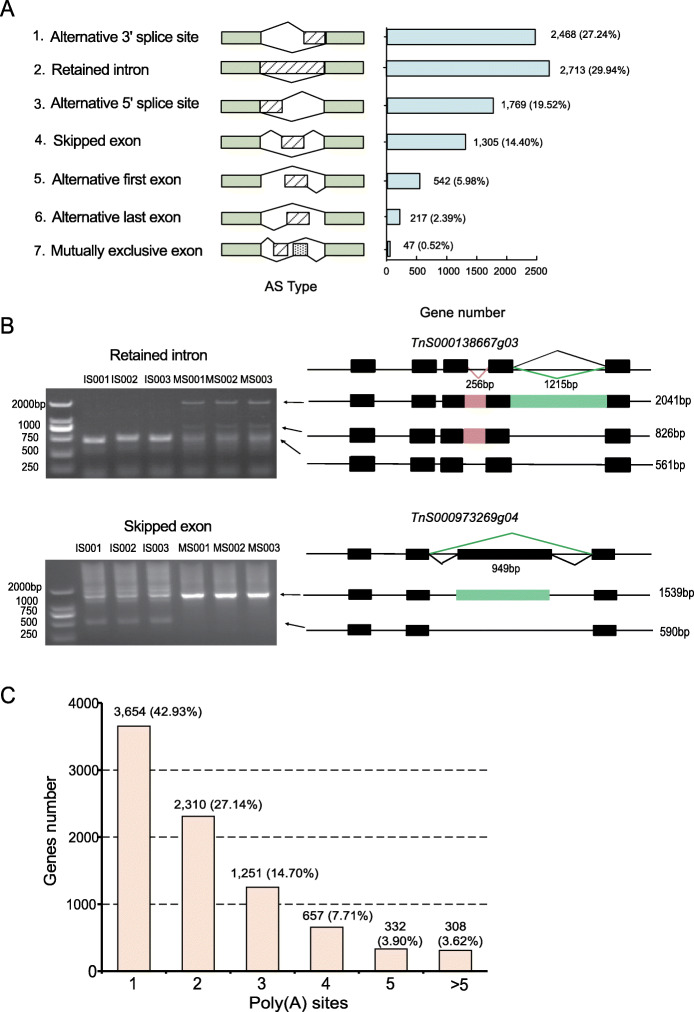
**a** Numbers of alternative splicing events identified in the full-length transcriptome of *G. luofuense*. **b** PCR validation of AS events, i.e. retained intention (at top) and skipped exon (at bottom) of two selected genes. **c** Genes with different numbers of alternative polyadenylation sites identified in the full-length transcriptome of *G. luofuense* seeds

After mapping reads to the reference genome of *G. luofuense*, a total of 9061 AS events were detected. These could be classified into seven types (Fig. 2a): retained intron (2713, 29.94%), alternative 3′ splice site (2468, 27.24%), alternative 5′ splice site (1769, 19.52%), skipped exon (1305, 14.40%), alternative first exon (542, 5.98%), alternative last exon (217, 2.39%), and mutually exclusive exon (47, 0.52%).

To verify the AS events identified, expression of two genes, i.e. *Tns00138667g03* and *TnS000973269g04* were validated by qRT-PCR (Fig. 2b, Additional file 1). In addition, a total of 8512 genes from *G. luofuense* seeds had at least one supported poly(A) site. Of these, 3654 (42.93%) had a single poly(A) site, and 640 (7.52%) had at least five poly(A) sites (Fig. 2c). The largest number of poly(A) sites—21—was found in the gene *TnS000670009g01*.

### Identification of TFs and lncRNAs

**Fig. 3:**
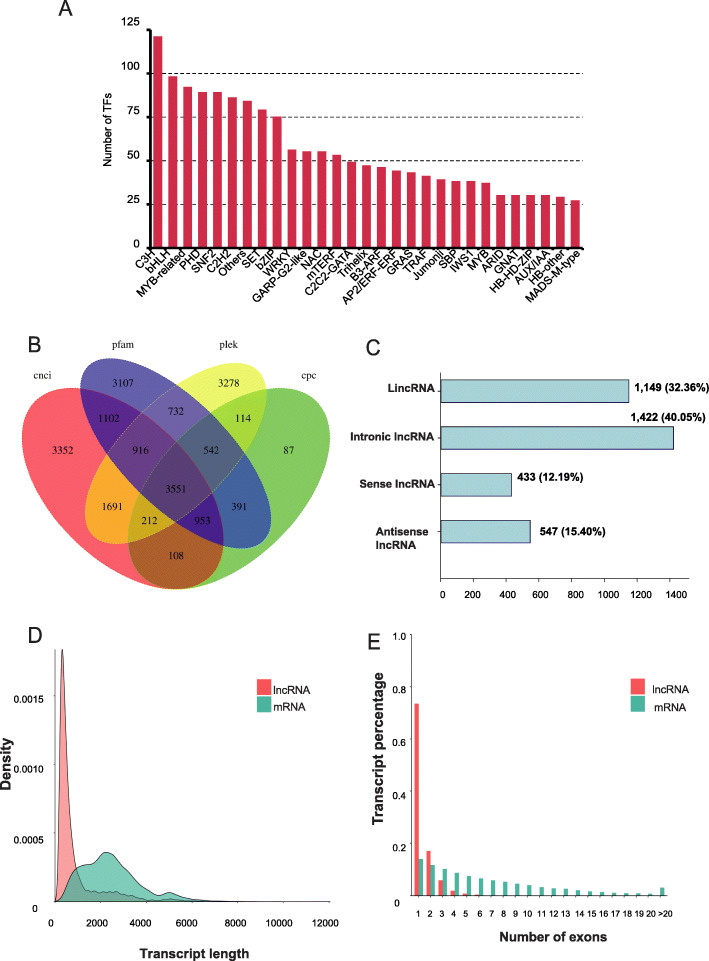
**a** A partial list of transcription factors (top 28 gene families) identified in the full-length transcriptome of *G. luofuense* seeds. **b** Venn diagram showing the number of lncRNAs identified using four different approaches: CPC (Coding Potential Calculator), CNCI (Coding-Non-Coding Index), CPAT (Coding Potential Assessment Tool), and Pfam (Protein Family). **c** Functional classification and numbers of four lncRNA types. **d** The length density distribution of identified lncRNAs on the reference genome of *G. luofuense* compared to that of identified lncRNA in the full-length transcriptome. **e** Distribution of exon numbers in mRNAs predicted by the reference genome and identified lncRNAs in the full-length transcriptome

A total of 2160 transcription factors (TFs) from 86 gene families were detected using iTAK. The largest fraction of identified TFs came from the C3H (5.6%), bHLH (4.53%), and MYB-related (4.26%) families (Fig. 3a). In addition, 11,885, 5958, 11,294 and 11,037 lncRNAs were identified using the CNCI, CPC, PFAM and PLEK methods, respectively. A total of 3551 lncRNAs were identified by all four methods (Fig. 3b**)**, with lengths ranging from 200 to 7840 bp. The lncRNAs were further classified into four types (Fig. 3c): 1422 (40.05%) sense intronic lncRNA, 1149 (32.36%) long intergenic non-coding RNA, 547 (15.40%) antisense lncRNA, and 433 sense overlapping lncRNA (12.19%). The length distribution of the identified lncRNAs was considerably narrower than that of mRNAs predicted from the *G. luofuense* genome (Fig. 3d). Moreover, most identified lncRNAs had five or fewer exons, whereas mRNAs predicted from the reference genome tended to have larger numbers of exons (Fig. 3e).

### Illumina sequencing of seed samples at two developmental stages

**Fig. 4:**
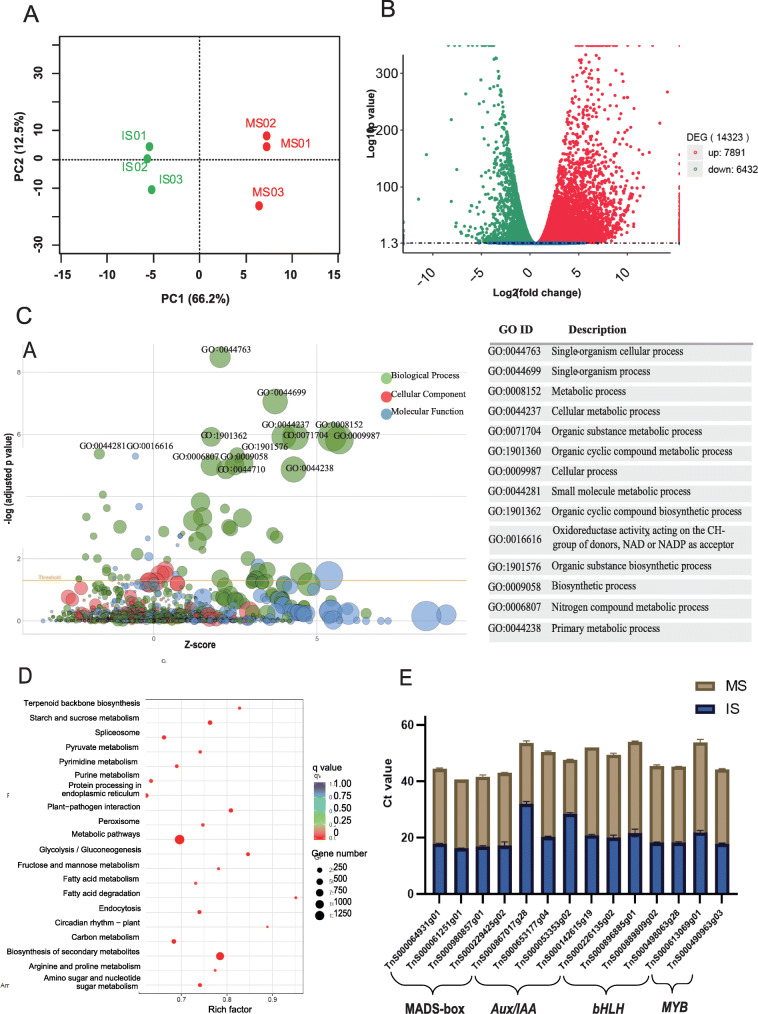
Detection of DEGs and qRT-PCR validation. **a** PCA analysis of gene expression in the three immature seed samples (IS01–03) and three mature seed samples (MS01–03). **b** A volcano plot of differential gene expression between immature and mature seed samples of *G. luofuense*, with upregulated genes in red and downregulated genes in green from immature seeds to mature seeds. **c** A bubble plot of enriched GO terms; the *x*-axis represents the *z*-score, the *y*-axis represents the negative logarithm of the adjusted *p-*values, the circle sizes are proportional to the number of genes enriched in the GO terms, and the circle colors denote the three GO term categories. A table on right side describes top 14 GO terms in the category biological process, which have been labeled in the bubble plot on left side. **d** A bubble plot of enriched KEGG terms; the *x*-axis represents rich factors, the circle sizes are proportional to enriched gene numbers, and the circle colors correspond to the negative logarithm of the adjusted *p*-values for each KEGG pathway. **e** The expression of 14 TF genes (i.e. MADS-box, *Aux/IAA*, *bHLH* and *MYB* genes) from immature and mature seeds of *G. luofuense* were verified by qRT-PCR, and the expression values were normalized with the △△Ct-method

To explore gene expression patterns during seed development of *G. luofuense*, 306,900,384 clean Illumina reads (46.04 Gb of raw data) with Q30 values from 93.54 to 94.07% were generated from three immature seed samples (IS) and three mature seed samples (MS) (Table S4). After the deletion of adaptors and low-quality reads, the average GC content of the six samples was 47.08%. PCA analysis showed that gene expression was highly correlated among the replicate samples of immature and mature seeds (correlation efficiency value = 0.95, cumulative proportion of variation explained by PC1 and PC2 = 78.7%) (Fig. 4a). After mapping to the *G. luofuense* genome, the mapping ratios of IS (average 89.44%, Table S5) were found to be significantly larger than those of MS (average 84.46%, Student’s *t*-test *p*-value = 0.003). RNA-seq analysis of the two developmental stages yielded a total of 23,977 genes (19,010 in IS and 20,737 in MS), of which 2970 were identified as novel genes.

### Enrichment analysis of DEGs and qRT-PCR validation

A total of 14,323 differentially expressed genes (DEGs) were identified between IS (control group) and MS: we found 7891 upregulated genes and 6432 genes downregulated (Fig. 4b) from IS to MS. The DEGs were also annotated with the three categories of GO terms, and multiple GO terms in the “biological process” category were significantly enriched with regard to *Z*-scores and adjusted *p*-values (Fig. 4c). The top five enriched GO terms were “single-organism cellular process” (GO:0044763), “single-organism process” (GO:0044699), “metabolic process” (GO:0008152), “cellular metabolic process” (GO:0044237), and “Organic substance metabolic process” (GO:0071704). The DEGs were also enriched in multiple KEGG pathways with reference to *Arabidopsis thaliana*. The top five enriched KEGG pathways were “metabolic pathways” (KEGG ID: ath01100, 1229 genes), “biosynthesis of secondary metabolites” (ath01110, 844 genes), “carbon metabolism” (ath01200, 179 genes), “ribosome” (ath03010, 164 genes), and “starch and sucrose metabolism” (ath00500, 154 genes) (Fig. 4d). qRT-PCR was used to validate the relative expression of 14 genes of interest: four MADS-box genes, four *Aux/IAA* genes, four *bHLH* genes, and two *MYB* genes. The relative expression of the 14 genes at the two seed developmental stages is presented in Fig. 4e.

## Discussion

### Structural analysis of the full-length transcriptome

#### Structural analysis of the full-length transcriptome

##### AS event analysis

In angiosperms, it has been reported that the percentages of AS modes differ dramatically among organs and that vegetative tissues (e.g. roots and ears) exhibit higher percentages of intron retention than reproductive tissues (e.g. pollen and endosperm) in maize (see Fig. 3 in 17). Moreover, retained intron percentage dramatically declines over the course of fruit development in strawberry [30]. In gymnosperms, *Gingko biloba* genes (e.g. *GB_12621* and *GB_20198*) show differences in AS between vegetative and reproductive organs and between immature and mature stages of leaves and seeds [26]. In the present study, retained intron accounted for 29.94% of all AS events (Fig. 2a), this figure was dramatically lower than those reported previously in leaves [41.5%, 12] and female strobili [46%, 11] of *G. luofuense*. This result suggests that the frequency of different AS modes may not only be species specific but may also vary among different organs.

##### APA analysis

In angiosperms, alternative polyadenylation is involved in the regulation of flowering time [31, 32]. In gymnosperms, it has been suggested that investigation of APA can improve genome annotation and promote understanding of flavonoid biosynthesis in *G. biloba* [26]. In the present study, a total of 8512 genes were identified as having at least one poly(A) site, the numbers of genes with various numbers of poly(A) sites declined dramatically as the number of poly(A) sites increased from one to five (Fig. 2c). The pattern of gene numbers with various numbers of poly(A) sites is consistent with that observed in *G. luofuense* leaves and female strobili [11]. Our results suggest that alternative polyadenylation enriches the proteomic complexity and affects the seed ripening process of *G. luofuense*.

##### lncRNA analysis

In angiosperms, lncRNAs participate in fruit development and color change in strawberry [33], as well as aroma formation in black tea [34]. In gymnosperms, lncRNAs have an important role in the regulation of leaf development [27] and leaf color changes in *G. biloba* [25]. The percentages of four lncRNA types have been shown to differ dramatically in *G. biloba*: lincRNA (50.6%), sense lncRNA (21.6%), intronic lncRNA (20.9%), and antisense lncRNA (6.9%) [26]. A recent study show that lincRNA was the highest (40.8%) and antisense lncRNA was the lowest (1.67%) in the full-length transcriptome of the *G. luofuense* female strobilus [11]. In the present study, however, we found number of intronic lncRNA (40.05%) was the highest, and that of sense lncRNA (2.1%) was the lowest (Fig. 3c). Thus, it appears that *G. luofuense* uses different lncRNAs to regulate the different reproductive organs. The scenario is similar to the results reported in *P. abies* [35]. Moreover, lncRNAs tended to be shorter and possessed fewer exons than protein coding genes [27], this finding is consistent with previous studies in gymnosperms, such as *G. biloba* [27], *Picea abies* [35], and *G. luofuense* [11].

### Key TFs/genes involved in seed ripening of *G. luofuense*

#### MADS-box genes

MADS-box transcription factors are classified into type I and type II groups based on the sequence of the conserved MADS domain [36, 37]. Compared with type II genes, type I genes have received less attention in previous studies, although their roles in the development of female gametophytes, embryos and seeds have been highlighted in angiosperms [36, 38]. The functions of type I genes are poorly understood in gymnosperms, and broad expression of type I genes in shoots, needles and strobili of conifers has been regarded as “transcriptional noise” [39]. Type I genes are further divided into Mα, Mβ and Mδ subgroups; Mα genes are generally expressed in various shoot tissues of conifers, whereas Mβ/Mδ genes are expressed in embryos, buds, and male strobili [39]. In *G. luofuense*, a total of 11 type I genes, (seven Mα genes, three Mβ genes, and one Mδ gene) have been reported [8]. Twenty-seven type I MADS-box TFs were identified in *G. luofuense* seeds (Fig. 4e), and gene *TnS000803113g11* was differentially expressed between immature and mature seeds, indicating an important role in seed ripening of *G. luofuense*.

Among type II MADS-box genes, the expression of *TM8* genes was first reported in tomato flowers [40] and the *TM8*-like gene ERAF17 was shown to be expressed in female flowers but not male flowers of cucumber [41]. In gymnosperms, *TM8*-like genes (e.g. *GbMADS11* and *GbMADS6* in *G. biloba* and *TbTM8* in *Taxus baccata*) influence aril development in male strobili and seed aril developing [42]. In *G. luofuense*, *TM8*-like genes accounted for almost half the number of genes with a varied pattern of expression [8]. For example, *TnS013912549g01* was expressed in both female and male strobili, whereas *TnS001008199t01* was exclusively expressed in male strobili [8]. A previous study showed that *GpMADS1*, a *TM8*-like gene defined in Hou et al. (2019b), participated in the development of female strobili in *G. parvifolium* [43]. Thus far, 38 type II genes have been identified in *G. luofuense*, of which *TM8*-like genes constitute almost half of the identified gene numbers [8]. In the present study, two *TM8*-like genes *TnS000061251g01* and *TnS000980857g01* were differentially expressed. Moreover, other type II MADS-box genes, such as *AG*-like gene *TnS000064931g01* and *AGL6*-like gene *TnS000229425g02* were differentially expressed at two developmental stages of *G. luofuense* seeds. Our results are congruent with a previous study that *AG*, *AGL6* and *TM8*-like genes regulate seed development of *G. biloba* and *T. baccata* [42].

#### Aux/IAA genes

*Aux/IAA* TFs play an essential role in the auxin responses of seed plants [9, 10, 44]. For example, in angiosperms, *FaAux/IAA1* and *Aux/IAA2* participate in the fruit development of strawberry [45], and *EgrIAA4* is thought to be essential to the regulation of secondary cell wall and fiber development in *Eucalyptus* [46]. Another study showed that *IAA9* was involved in fruit and leaf morphogenesis in tomato [47] In gymnosperms, the *Aux/IAA* gene *LaIAA2* appears to be important for the regulation of root development and auxin signaling [48]. Besides, six *Aux/IAA* genes (*GluIAA1*–*6*) have been identified in *G. luofuense*, all of which are involved in female strobilus development [11]. In the present study, four *Aux/IAA* genes, *TnS000653177g04* (*GluIAA2*), *TnS000867017g28* (*GluIAA3*), *TnS000053353g02* (*GluIAA4*), and *TnS000142615g19* (*GluIAA5*), were differntially expressed and validated by qRT-PCR between the two developmental stages of *G. luofuense* seeds (Fig. 4e). These results suggest that *Aux/IAA* genes may also be of importance in *G. luofuense* seed ripening.

#### bHLH genes

In angiosperms, the bHLH-encoding gene *SPATULA* has been reported to control the development of flowers and fruits in *Arabidopsis* [49, 50], and a bHLH TF has been shown to determine seed coat color in *Brassica rapa* [51]. Moreover, *bHLH* TFs, together with MYB and WDR TFs, are involved in the regulation of flavonoid biosynthesis [52–54]. The expression of two *MYB*-related genes, i.e. *Osmyb1* and *Osmyb4*, reaches the level of saturation at 14 days after the anthesis, suggesting that they have an important role in the maturation of rice seeds [55]. In gymnosperms, three bHLH TFs have been reported to negatively regulate gene expression in the paclitaxel biosynthesis pathway in response to jasmonate in *Taxus cuspidata* [56]. Besides, bHLH and MYB TFs have been reported to participate in flavonoid biosynthesis in the roots rather than the seeds of *Ginkgo biloba* [26]. In *G. luofuense*, 67 *bHLH* genes were identified in leaves based on full-length transcripts; 30 were subjected to phylogenetic analysis and classified into four subgroups [57]. Furthermore, 110 bHLH TFs were the most abundant TFs during development of the female strobilus in *G. luofuense* [11]. In the present study, 98 bHLH were identified, of which four bHLH genes, i.e. *Tns000226135g02*, *TnS000896885g01*, *TnS000889809g02*, and *TnS000498063g28* were differentially expressed and their expression was validated by qRT-PCR (Fig. 4e). These results suggest that bHLH TFs may also play an imporant role in seed ripening of *G. luofuense*.

#### Genes related to carbohydrate metabolism

*Gnetum* seeds are rich in carbohydrates as the examples reported in *G. africanum* (87.62%) [5] and *G. gnemon* (64.1%) [6]. The accumulation of carbohydrates in *Gnetum* seeds makes them palatable and nutritious, thereby attracting a variety of herbivores to promote seed dispersal [58, 59]. In the present study, the DEGs between immature and mature seeds were enriched in several KEGG pathways, e.g. carbon metabolism, starch and sucrose metabolism, glycolysis/gluconeogenesis, and fructose and mannose metabolism (Fig. 4d). The DEGs were also enriched in the GO terms, e.g. primary metabolic process, metabolic process, and cellular metabolic process (Fig. 4c). These results suggest that genes that are involved in carbohydrate metabolisms are also indispensable in seed ripening of *G. luofuense*.

## Conclusions

We generated a full-length transcriptome of *G. luofuense* seeds at two developmental stages using Pacbio sequencing technique. We identified a total of 5726 novel genes, 9061 alternative splicing events, 3551 lncRNAs, and 8512 genes were identified to possess at least one poly(A) site. Transcription factors MADS-box, Aux/IAA and bHLH were found to play important roles in seed ripening of *G. luofuense*. These findings provide a valuable molecular resource for disentangling organ development of gymnosperms.

## Methods

### Plant material and RNA extraction

*Gnetum luofuense* seeds were collected at immature (IS) and mature (MS) developmental stages from a female individual (voucher number “CH003”, SYS) cultivated in the Bamboo Garden at Sun Yat-sen University on September 2nd and 28th 2018 (Fig. 1a) with the permissions of Sun Yat-sen University. To obtain a full-length transcriptome for the two developmental stages, identical amounts (10 g) of mature and immature seeds with arils were pooled, incubated in liquid nitrogen, and frozen at − 20 °C for PacBio SMRT sequencing. In addition, six samples of *G. luofuense* seeds (“IS001–003” and “MS001–003”) were collected for Illumina sequencing, three from the immature stage (control group) and three from the mature stage. The RNA for each sample was extracted using an RNA kit (Qiagen, Valencia, CA, USA) following the manufacturer’s instructions. RNase-free DNase (Qiagen) was used to remove relic DNA, and the RNA concentration of samples was evaluated by 1% agarose gel electrophoresis. A NanoDrop spectrophotometer (ThermoFisher Scientific, Wilmington, DE, USA) and Agilent 2100 Bioanalyzer (Agilent Technologies, Palo Alto, CA, USA) were used to assess the purity and integrity of the extracted RNA. *G. luofuense* samples used in this research is derived from the plant cultivated merely for teaching and researches. Thus, the collection of seeds and the performance of experimental research on such plant were complied with the national guidelines of China.

### Plant material and RNA extraction

*Gnetum luofuense* seeds were collected at immature (IS) and mature (MS) developmental stages from a female individual (voucher number “CH003”, SYS) cultivated in the Bamboo Garden at Sun Yat-sen University on September 2nd and 28th 2018 (Fig. 1a). To obtain a full-length transcriptome for the two developmental stages, identical amounts (15 g) of mature and immature seeds with arils were pooled, incubated in liquid nitrogen, and frozen at − 20 °C for PacBio SMRT sequencing. In addition, the six samples of *G. luofuense* seeds were collected for Illumina sequencing, three from the immature stage (control group) and three from the mature stage. The RNA for each sample was extracted using an RNA kit (Qiagen, Valencia, CA, USA) following the manufacturer’s instructions. RNase-free DNase (Qiagen) was used to remove relic DNA, and the RNA concentration of samples was evaluated by 1% agarose gel electrophoresis. A NanoDrop spectrophotometer (ThermoFisher Scientific, Wilmington, DE, USA) and Agilent 2100 Bioanalyzer (Agilent Technologies, Palo Alto, CA, USA) were used to assess the purity and integrity of the extracted RNA.

### Library construction and PacBio sequel sequencing

When the integrity of extracted RNA met the minimum requirement (> 7.0), full-length cDNA was synthesized using a SMARTer PCR cDNA Synthesis kit (Clontech, Takara Bio Inc., Shiga, Japan). The synthesized cDNA was subjected to PCR amplification using a KAPA HIFI PCR kit (Kapa Biosystems, Boston, MA, USA). After PCR amplification, the cDNA was quality controlled and purified using a QIAquick PCR Purification kit (Qiagen, Hilden, Germany). The RNA samples were subjected to terminal repair and the attachment of SMRT dumbbell-type adapters. Before PacBio sequencing, two bins (1–4 kb, 4–6 kb) were established to preferentially sequence the smaller cDNAs.

### Library construction and Illumina sequencing

Before Illumina sequencing, all six RNA samples that possessed poly(A) were enriched with oligo (dT) magnetic beads. The enriched RNA was randomly reduced to small pieces with a fragmentation buffer. First strand cDNA was generated using hexamers and reverse transcriptase (Superscript III, Invitrogen). After purification with AMPure XP beads, second strand cDNA was synthesized using DNA polymerase I, RNase H and dNTPs (Sigma-Aldrich). The double-stranded cDNA was subjected to terminal repair and poly(A) tailing, followed by Illumina adaptor ligation. The final cDNA library was completed after a second round of purification and PCR amplification. The quality of the six cDNA libraries was assessed using a Qubit 2.0 fluorometer prior to sequencing on the Illumina HiSeq 4000 platform.

### PacBio data processing and error correction

PacBio sequencing data were analyzed using PacBio SMRTlink v. 5.1 software. First, we obtained reads of inserts (ROIs) from the BAM files generated from the platform using the following parameters: maximum drop fraction—0.8, minimum length—200, no polish, minimum *z*-score—9999, minimum passes—1, minimum predicted accuracy—0.8, and maximum length—18,000. The ROIs were classified into full-length reads (FLs) and non-full-length reads (nFLs) based on the presence and absence of 5′ and 3′ cDNA primers and a 3′ poly (A) tail, see also in [11]. The FLs and nFLs were clustered to achieve consensus isoforms using an isoform-level clustering (ICE) algorithm. To obtain full-length non-chimeric (FLNC) isoforms, the high-quality isoforms from FLs were corrected using Quiver software with a post-correction accuracy above 99%. The low-quality consensus isoforms from nFLs were further corrected with LoRDEC [60] using two Illumina-sequenced samples (one from mature seeds and one from immature seeds).

### Genome mapping and novel gene detection

All FLNCs and corrected nFLs were mapped to the reference genome of *G. luofuense* (=*G. montanum*) [29] using GMAP [61]. The GMAP output files were used for subsequent analyses. Redundant FLNCs were removed using the following parameters: minimum identity—0.9, minimum trimmed coverage—0.85, and allow close indel—0. Mapped FLNCs with different lengths at their 5′ ends were not considered to be redundant. The FLNCs that mapped to annotated genes in the *G. luofuense* genome were considered to be known genes; otherwise, they were classified as novel genes and novel isoforms of known genes.

### Functional annotation and classification

All identified novel genes were annotated by BLASTX v.2.2.26 searches (E-value < 1 × 10^− 5^) of the gene ontology (GO, http://www.geneontology.org), Kyoto Encyclopedia of Genes and Genomes (KEGG, http://www.genome.jp/kegg/), Protein Family (Pfam), KOG/COG (Clusters of Orthologous Groups of proteins, http://www.ncbi.nlm.nih.gov/COG/), NCBI non-redundant protein sequence (NR, http//www.ncbi.nlm.nih.gov/), and Swiss-Prot (http// http://www.expasy.org/sprot/) databases and by HMMER v.3.1b2 searches (E-value < 1 × 10^− 10^) of the Pfam (Protein Family, http://pfam.xfam.org/) database [62, 63]. In addition, GO enrichment analysis was performed using the GOseq package implemented in R [64] and KEGG enrichment analysis was performed using KOBAS version 2.0 [65].

### AS and APA analysis

Gene structure analysis was performed using the TAPIS pipeline [16]. First, seven types of alternative splicing (AS) events were identified: alternative 3′ splice site, retained introns, alternative 5′ splice site, skipped exon, alternative first exon, alternative last exon, and mutually exclusive exons. In addition, alternative polyadenylation (APA) analysis was conducted, and genes were classified according to their poly(A) number.

### Identification of TFs and lncRNAs

Coding sequences (CDS), which possess open reading frames (ORFs), were identified by searching against the Pfam database using TransDecoder [66]. Based on the identified CDS, transcription factors (TFs) were predicted by searching against the Plant Transcription Factor Database v.4.0 (http://planttfdb.cbi.pku.edu.cn) using iTAK version 15.03 [67]. Four methods were used to identify lncRNAs: PC (Coding Potential Calculator), CNCI (Coding-Non-Coding Index), CPAT (Coding Potential Assessment Tool), and Pfam. The lncRNAs, which are longer than 200 nt and possess at least two exons, do not encode proteins and are classified into four groups: lincRNA, intronic lncRNA, sense lncRNA, and antisense lncRNA.

### DEG identification and qRT-PCR validation

Illumina sequenced raw reads with poly(N) and low scores were removed, and the remaining reads were trimmed of adaptors at both ends. The cleaned reads were mapped to the *G. luofuense* genome using HISAT2 v.2.1.0 [68]. Mapped read numbers were counted and adjusted through one scaling normalized factor using the R package edgeR [69]. The numbers of mapped reads were converted to values of fragments per kilobase of transcript per million mapped fragments (FPKM). To identify differentially expressed genes (DEGs), RNA data from three replicate samples of mature and immature seeds were separately merged and then compared using the R package EBSeq v. 1.20.0 [70]. The DEGs met the following requirements: corrected *P*-value (adjusted by the Benjamini & Hochberg method)—0.005 and log_2_(fold change) value—1.

### qRT-PCR validation

To validate the occurrence of AS events identified in the full-length transcriptome, expression of two genes, i.e. *TnS000138667g03* and *TnS000973269g04* were validated by qRT-PCR. Among the two micrograms of RNA were extracted from mature and immature seeds of *G. luofuens*e and subjected to cDNA synthesis according to the manufacturer’s protocol. qRT-PCR was performed under the following conditions: 10 min at 95 °C (1 cycle), 10 s at 95 °C, 30 s at 55 °C and 15 s at 72 °C (40 cycles), temperature reduction from 95 °C to 60 °C (0.5 °C/10 s) and termination in 30 s at 25 °C. Gene electrophorese was performed to test various lengths of qRT-PCR products. Moreover, 14 DEGs were selected for gene expression validation with qRT-PCR. The *G. luofuense* actin gene was used as an endogenous control to estimate the relative expression of target genes using the ΔΔCt-method [71]. For each sample, three replicates were performed, and the mean and standard deviation of the qRT-PCR gene expression values were calculated accordingly.

## Supplementary Information

**Additional file 1:** **Fig. S1.** Statistics and quality control of full-length transcripts. **a** Length distribution of subreads. **b** Length distribution of FLNCs. **c** length distribution of consensus isoforms using an isoform-level clustering algorithm. **d** Statistics and classification of full-length transcripts against the *G. luofuense* reference genome. **e** Mapping read density on the *G. luofuense* scaffolds; the *x*-axis represents the scaffold position (Mb), the *y*-axis represents the median read density (log2), and the green and red lines represent the positive and negative strands of the scaffolds, respectively. **f** The scale and identity range of all mapped full-length transcripts. The red and blue bars represent the coverage and identity of full-length reads, respectively.

**Additional file 2:** **Fig. S2.** Annotation summary of novel genes from *G. luofuense* seeds. **a** The distribution of NR annotations among different seed plant species, the *x*-axis represents the number of annotated reads. **b** KEGG enrichment of the annotated novel genes, the *x*-axis represents the number of annotated reads. **c** Gene ontology (GO) annotation and categorization of full-length transcripts.

**Additional file 3:** **Table S1.** Detail information in the processing of PacBio sequencing data.

**Additional file 4:** **Table S2.** Detail information in PacBio sequencing data corrected by Illumina sequencing data.

**Additional file 5:** **Table S3.** Summary of annotated numbers of novel genes by the six databases.

**Additional file 6:** **Table S4.** Detail information of Illumina sequenced data from the six *G. luofuense* seed samples.

**Additional file 7:** **Table S5.** Detail information of genome mapping of the transcriptome from the six *G. luofuense* seed samples.

## Data Availability

PacBio sequencing data from the merged seed sample and RNA-seq data from the six samples were all deposited in the NCBI Sequence Read Archive (SRA) under BioProject accession number PRJNA622631. The data that support the results are included within the article and its additional files. Other relevant materials are available from the corresponding authors on reasonable request.

## Abbreviations

DEGs: Differentially expressed genes
CDS: Coding sequences
ORFs: Open reading frames
TFs: Transcription factors
PC: Coding potential calculator
CNCI: Coding-non-coding index
CPAT: Coding potential assessment tool
APA: Alternative polyadenylation
FLs: Full-length reads
nFLs: Non-full-length reads
Aux/IAA: Auxin/indole-3-acetic acid
bHLH: Basic helix-loop-helix
