# Supplementary figures and images for: A full-length transcriptome and gene expression analysis reveal genes and molecular elements expressed during seed development in Gnetum luofuense
Source: BMC Plant Biol. 2020 Nov 23;20:531. doi: 10.1186/s12870-020-02729-1 (PMC7685604; doi:10.1186/s12870-020-02729-1)

A

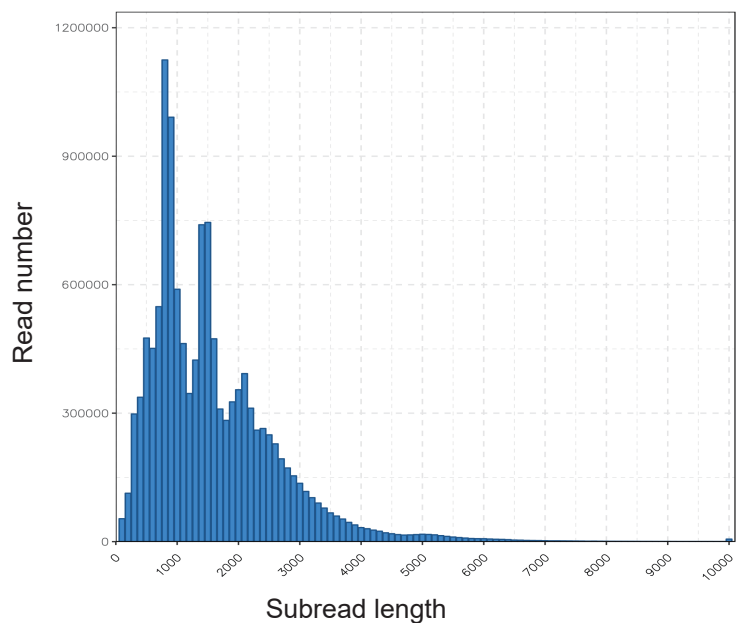

B

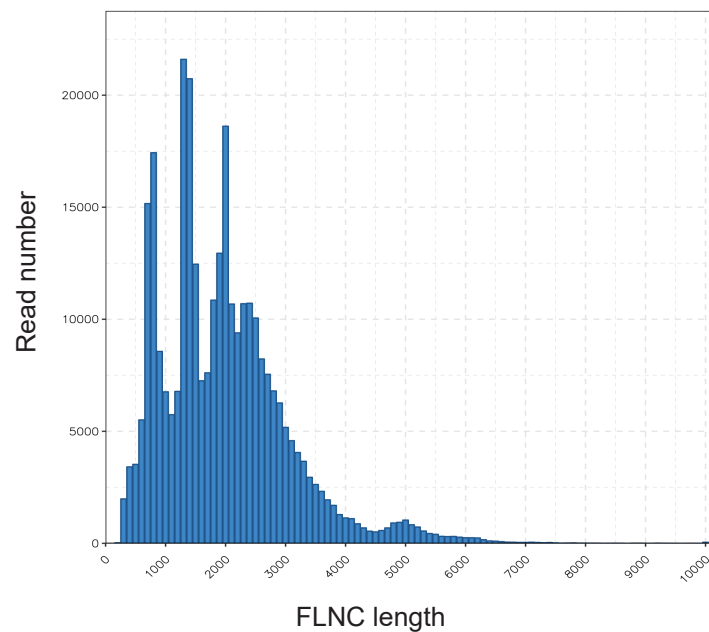

C

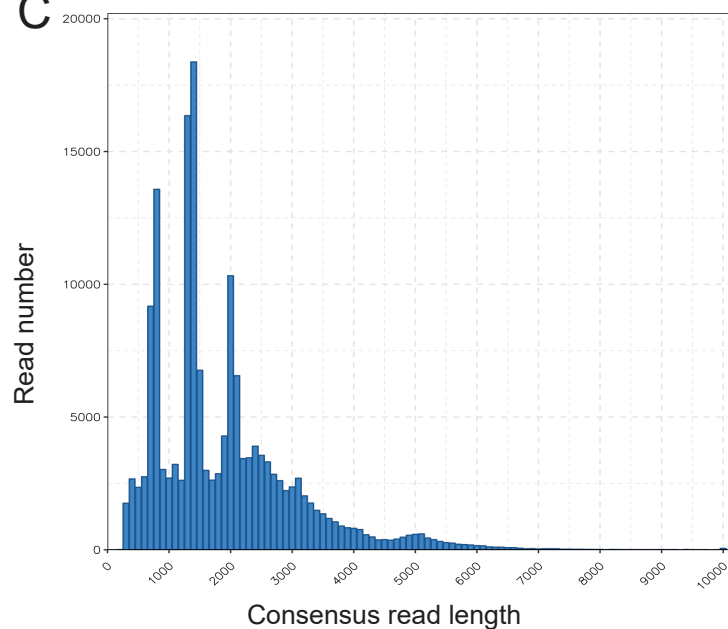

D

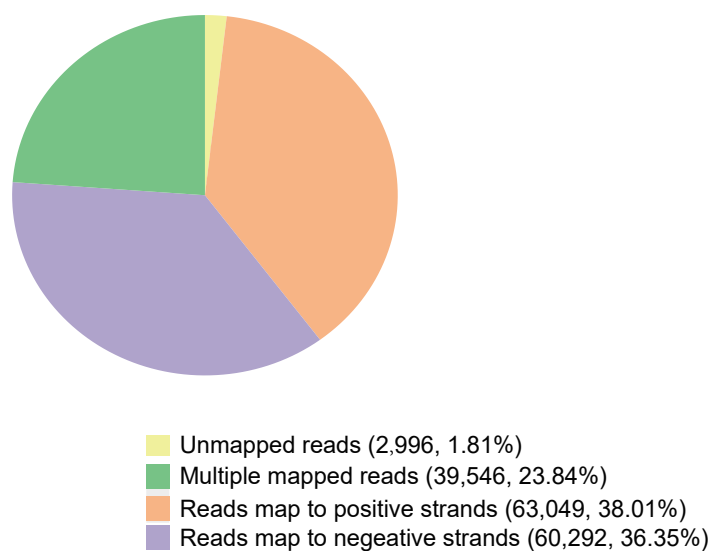

E

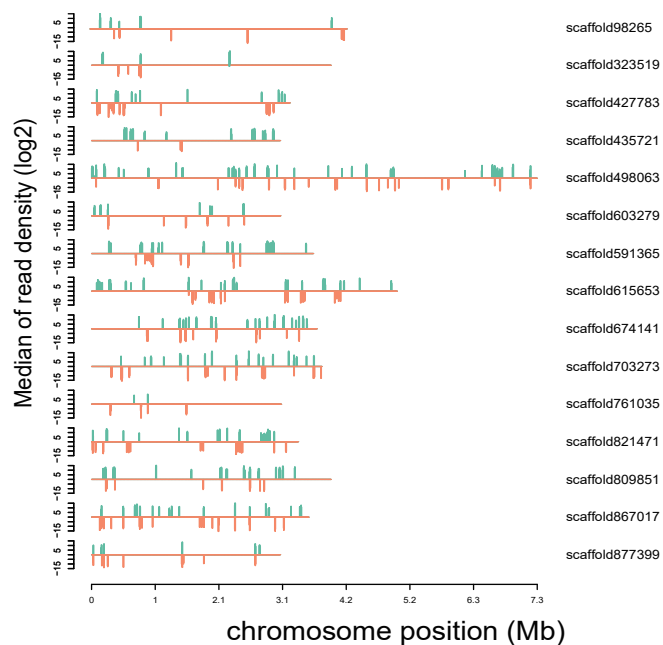

F

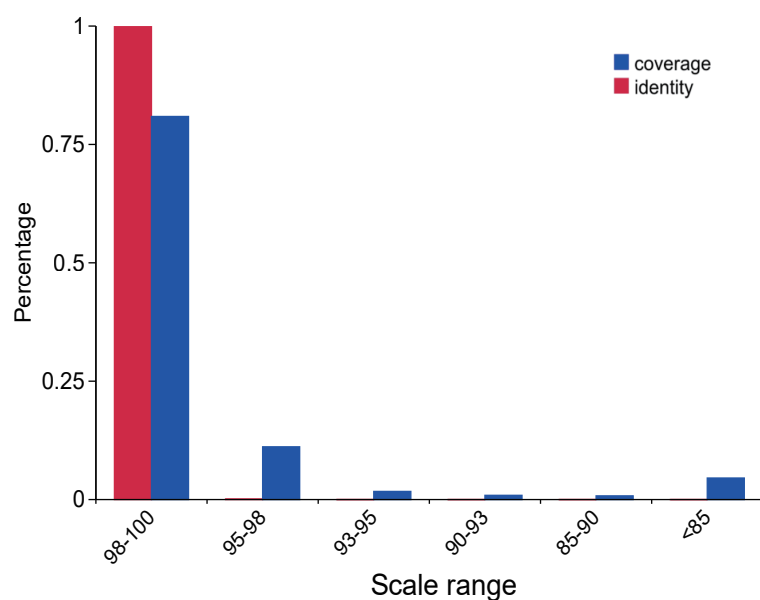

Supplement: Supplementary file 1 — Additional file 1: Fig. S1. Statistics and quality control of full-length transcripts. a Length distribution of subreads. b Length distribution of FLNCs. c length distribution of consensus isoforms using an isoform-level clustering algorithm. d Statistics and classification of full-length transcripts against the G. luofuense reference genome. e Mapping read density on the G. luofuense scaffolds; the x-axis represents the scaffold position (Mb), the y-axis represents the median read density (log2), and the green and red lines represent the positive and negative strands of the scaffolds, respectively. f The scale and identity range of all mapped full-length transcripts. The red and blue bars represent the coverage and identity of full-length reads, respectively. [file 12870_2020_2729_MOESM1_ESM.pdf]

A

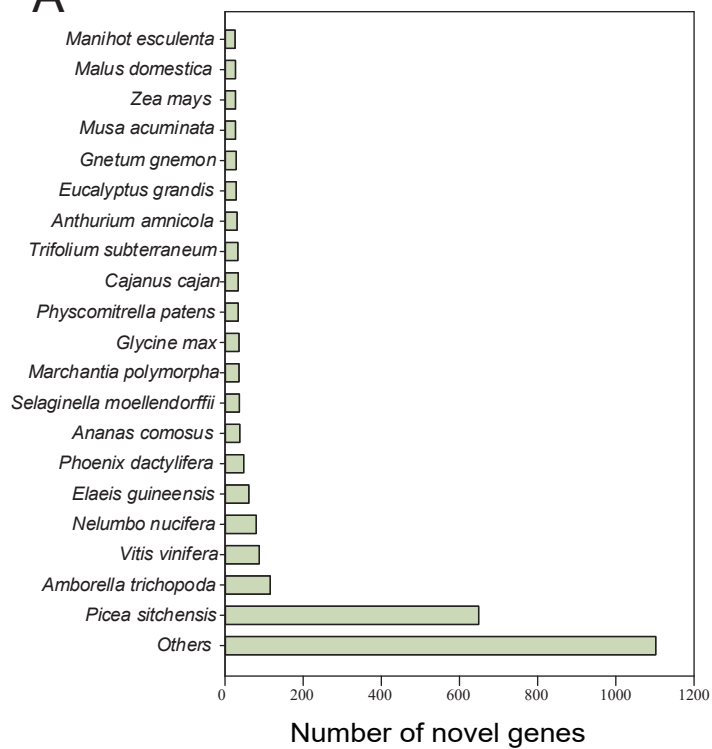

B

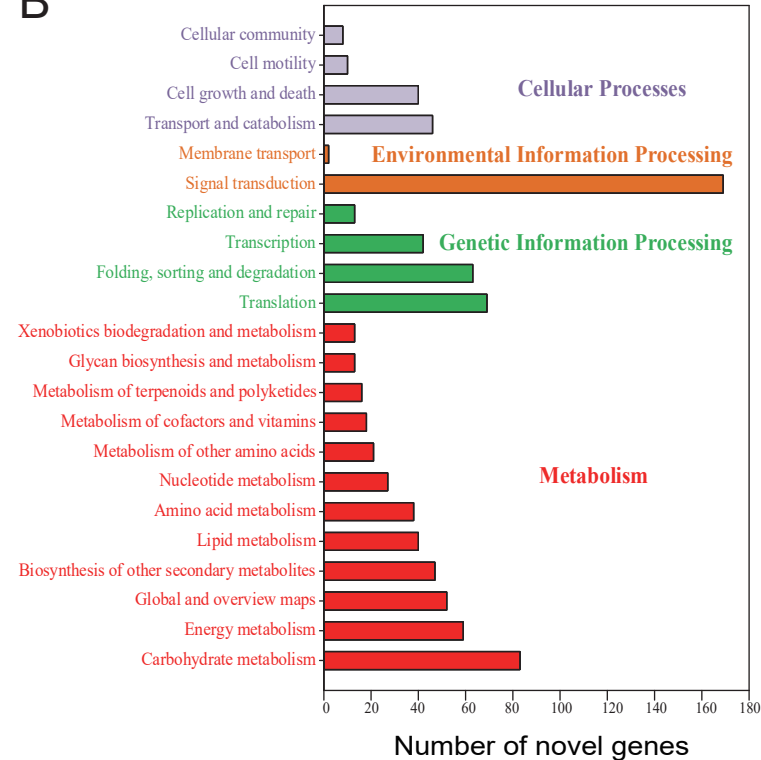

C

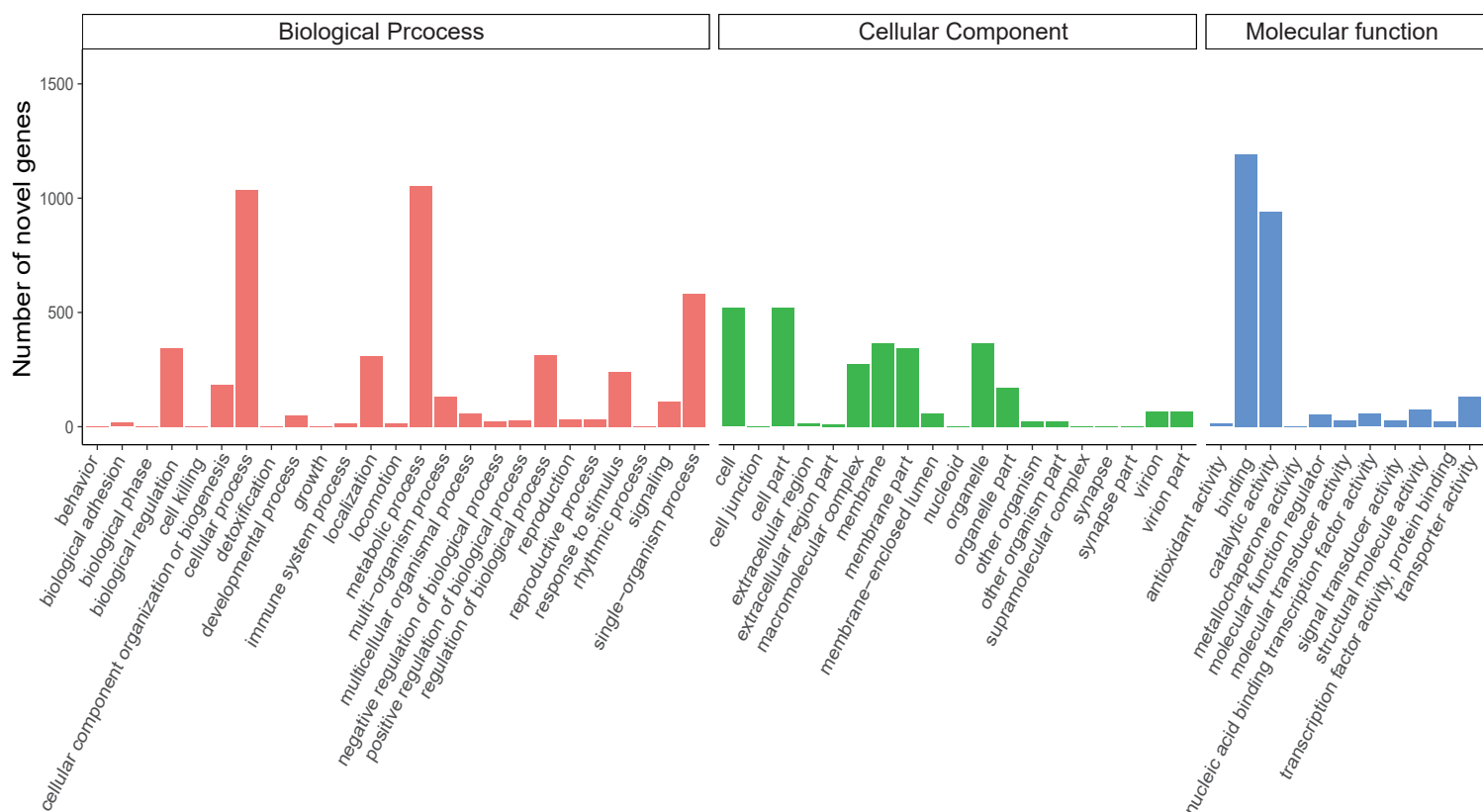

Supplement: Supplementary file 2 — Additional file 2: Fig. S2. Annotation summary of novel genes from G. luofuense seeds. a The distribution of NR annotations among different seed plant species, the x-axis represents the number of annotated reads. b KEGG enrichment of the annotated novel genes, the x-axis represents the number of annotated reads. c Gene ontology (GO) annotation and categorization of full-length transcripts. [file 12870_2020_2729_MOESM2_ESM.pdf]
