# Supplementary material for: A full-length transcriptome and gene expression analysis reveal genes and molecular elements expressed during seed development in Gnetum luofuense
Source: BMC Plant Biol. 2020 Nov 23;20:531. doi: 10.1186/s12870-020-02729-1 (PMC7685604; doi:10.1186/s12870-020-02729-1)
Supplement: Supplementary file 3 — Additional file 3: Table S1. Detail information in the processing of PacBio sequencing data. [file 12870_2020_2729_MOESM3_ESM.docx]

**Table S1. Detail information in the processing of PacBio sequencing data**

| **Terms** | **Numbers or ratio** |
| --- | --- |
| Subreads base (G) | 19.81 |
| Number of subread | 12,869,707 |
| Average length of subread | 1,540 |
| N50 of subread | 2,013 |
| Number of CCS | 384,042 |
| Number of sequences with 5 'terminal primer | 362,429 |
| Number of sequences with 3 'terminal primer | 362,580 |
| Number of sequences with poly(A) tail | 335,541 |
| Number of full length sequence | 317,094 |
| Number of full-length non-chimeric reads (Flnc) | 312,444 |
| Average length of Flnc read | 1,919 |
| Percentage of Flnc (%) | 0.81 |
| Number of polished consensus read | 165,883 |
| Minimum length of consensus read | 167 |
| Maximum length of consensus read | 13,816 |
| Average length of consensus read | 1,847 |
| N50 of consensus read | 2,245 |
