# Supplementary material for: A full-length transcriptome and gene expression analysis reveal genes and molecular elements expressed during seed development in Gnetum luofuense
Source: BMC Plant Biol. 2020 Nov 23;20:531. doi: 10.1186/s12870-020-02729-1 (PMC7685604; doi:10.1186/s12870-020-02729-1)
Supplement: Supplementary file 4 — Additional file 4: Table S2. Detail information in PacBio sequencing data corrected by Illumina sequencing data. [file 12870_2020_2729_MOESM4_ESM.docx]

**Table S2. Detail information in PacBio sequencing data corrected by Illumina sequencing data**

| **Type** | **Before correction** | **After correction** |
| --- | --- | --- |
| Total nucleotide | 306,359,206 | 307,446,027 |
| Total sequence | 165,883 | 165,883 |
| Mean length | 1,847 | 1,854 |
| Minimum length | 167 | 155 |
| Maximum length | 13,816 | 14,509 |
| N50 | 2,245 | 2,254 |
| N90 | 1,175 | 1,179 |
