# Supplementary material for: A full-length transcriptome and gene expression analysis reveal genes and molecular elements expressed during seed development in Gnetum luofuense
Source: BMC Plant Biol. 2020 Nov 23;20:531. doi: 10.1186/s12870-020-02729-1 (PMC7685604; doi:10.1186/s12870-020-02729-1)
Supplement: Supplementary file 5 — Additional file 5: Table S3. Summary of annotated numbers of novel genes by the six databases. [file 12870_2020_2729_MOESM5_ESM.docx]

**Table S3. Summary of annotated numbers of novel genes by the six databases**

| **Databases** | **Novel gene numbers** |
| --- | --- |
| GO | 2,069 |
| KEGG | 2,487 |
| KOG | 1,315 |
| NR | 2,588 |
| Pfam | 2,069 |
| SwissProt | 1,930 |
| Total annotated genes | 4,099 |
