# Supplementary material for: A full-length transcriptome and gene expression analysis reveal genes and molecular elements expressed during seed development in Gnetum luofuense
Source: BMC Plant Biol. 2020 Nov 23;20:531. doi: 10.1186/s12870-020-02729-1 (PMC7685604; doi:10.1186/s12870-020-02729-1)
Supplement: Supplementary file 6 — Additional file 6: Table S4. Detail information of Illumina sequenced data from the six G. luofuense seed samples. [file 12870_2020_2729_MOESM6_ESM.docx]

**Table S4. Detail information of Illumina sequenced data from the six *G. luofuense* seed samples**

| **Sample name** | **Raw reads** | **Clean reads** | **Q20 (%)** | **Q30 (%)** | **GC content (%)** | **Genome mapping (%)** |
| --- | --- | --- | --- | --- | --- | --- |
| IS01 | 55,631,598 | 54,243,040 | 97.77 | 93.55 | 47.12 | 89.75 |
| IS02 | 49,049,534 | 47,624,272 | 97.99 | 94.07 | 46.93 | 88.74 |
| IS03 | 59,174,882 | 57,466,054 | 97.76 | 93.54 | 47.56 | 89.82 |
| MS01 | 54,240,794 | 53,255,114 | 97.86 | 93.88 | 46.98 | 85.32 |
| MS02 | 52,496,962 | 50,708,568 | 97.85 | 93.78 | 46.71 | 84.57 |
| MS03 | 44,723,634 | 43,603,336 | 97.82 | 93.64 | 47.18 | 83.49 |
| Total | 315,317,404 | 306,900,384 | 97.84 | 93.74 | 47.08 | 86.95 |
