# Supplementary material for: A full-length transcriptome and gene expression analysis reveal genes and molecular elements expressed during seed development in Gnetum luofuense
Source: BMC Plant Biol. 2020 Nov 23;20:531. doi: 10.1186/s12870-020-02729-1 (PMC7685604; doi:10.1186/s12870-020-02729-1)
Supplement: Supplementary file 7 — Additional file 7: Table S5. Detail information of genome mapping of the transcriptome from the six G. luofuense seed samples. [file 12870_2020_2729_MOESM7_ESM.docx]

**Table S5. Detail information of genome mapping of the transcriptome from the six *G. luofuense* seed samples**

| **Sample name** | **IS01** | **IS02** | **IS03** | **MS01** | **MS02** | **MS03** |
| --- | --- | --- | --- | --- | --- | --- |
| Total reads | 54,243,040 | 47,624,272 | 57,466,054 | 53,255,114 | 50,708,568 | 43,603,336 |
| Total mapped | 48,642,362 (89.67%) | 42,217,664 (88.65%) | 51,570,801 (89.74%) | 45,412,254 (85.27%) | 42,897,372 (84.6%) | 36,405,206 (83.49%) |
| Multiple mapped reads | 1,210,358 (2.23%) | 1,063,787 (2.23%) | 1,296,609 (2.26%) | 1,589,381 (2.98%) | 1,416,262 (2.79%) | 1,337,971 (3.07%) |
| Uniquely mapped reads | 47,432,004 (87.44%) | 41,153,877 (86.41%) | 50,274,192 (87.49%) | 43,822,873 (82.29%) | 41,481,110 (81.8%) | 35,067,235 (80.42%) |
| Reads map to '+' strands | 23,746,949 (43.78%) | 20,608,975 (43.27%) | 25,166,379 (43.79%) | 21,941,014 (41.2%) | 20,771,109 (40.96%) | 17,543,656 (40.23%) |
| Reads map to '-' strands | 23,685,055 (43.66%) | 20,544,902 (43.14%) | 25,107,813 (43.69%) | 21,881,859 (41.09%) | 20,710,001 (40.84%) | 17,523,579 (40.19%) |
